# Editorial: Keep Calm and Care for Your Microbiota: The Role of *H. pylori* and Microbiota in Gastric Diseases

**DOI:** 10.3389/fmicb.2021.692472

**Published:** 2021-07-14

**Authors:** Rossella Grande, Guillermo I. Perez Perez

**Affiliations:** ^1^Department of Pharmacy, University “G. d'Annunzio” of Chieti-Pescara, Chieti, Italy; ^2^Department of Medicine New York University Langone Medical Center, New York, NY, United States

**Keywords:** *Helicobacter pylori*, virulence factors, microbial dysbiosis, biofilm, outer membrane vesicles, probiotics, VBNC state, gastric microbiota/microbiome

*Helicobacter pylori* is a fastidious, microaerophilic, pleomorphic microorganism that colonizes the stomach of nearly half of the world's human population. It was isolated for the first time almost 39 years ago and is the etiological agent of many gastric diseases such as gastritis, gastric and duodenal ulcer, and gastric mucosa-associated lymphoid tissue (MALT) lymphoma. In addition, *H. pylori* has been recognized as the main risk factor for the development of distal gastric cancer (GC), the most common malignancy worldwide that has a high mortality rate (Li and Perez-Perez, 2018). Major progress has been made in preventing some of the diseases in which *H. pylori* is the main etiological agent. However, some major challenges in the treatment and control of *H. pylori* infection remain after all these years, one is to reduce the risk of gastric carcinoma and the second one is to improve the efficacy of eradication therapies.

Distal gastric cancer associated with *H. pylori* is multifactorial. The presence of virulence factors, severe chronic inflammation, and the production of oxidative stress factors by the host have been associated with GC development, in addition to environmental and dietary factors (Alarcón et al., 2017; Piscione et al.). More recently it has been described that dysbiosis of the gut microbiota also play a role in worsening the prognosis in the *H. pylori*-infection. Numerous studies demonstrated that colonization of the stomach by *H. pylori* affected the composition of the gastric microbiota associated with modulating the acidity of the stomach and, as a result of this, promote the development of gastric diseases and in particular gastric cancer (Alarcón et al., 2017; Piscione et al.). The development of GC may be due to a possible interplay between the resident microbes, environment, and host immune response. The novel technology of sequencing analysis has increased our knowledge of the microbiome in humans and animals in different anatomic sites and confirmed that modifications in the composition of the gut microbiome might play a key role in the development of severe gastric diseases. Several studies have demonstrated the carcinogenic role of *H. pylori* in the development of premalignant lesions. In addition, some authors have demonstrated that the gastric colonization of non-*H. pylori* bacteria also represents an additional risk factor for the development of gastric cancer, unfortunately, little data is available on the potential carcinogenic role of non-*H. pylori* and their metabolites (Yang et al.). Differences in the microbial profile and composition between early gastric cancer (EC) and advanced gastric cancer (AC), have been demonstrated and confirmed alterations associated with gastric cancer progression. The analysis of gastric microbiota demonstrated that the production of urease and synthesis of bacterial flagella were lower in EC, in contrast, the glycolysis of fructose and hydrolysis of glycosides was increased. The identified microbial signature reported by Wang et al. might represent a useful biomarker for the clinical assessment of gastric cancer risk. The advances associated with the role of the gut microbiota during gastric carcinogenesis, in particular, those focalized on the identification of the bacterial taxa colonizing the gastric mucosa might help to predict the outcome of gastric diseases by preventing the development of gastric carcinoma and providing important information to manage the infection with appropriate therapy.

Numerous epidemiological studies have demonstrated that the eradication of *H. pylori* is associated with a decrease in diseases related to *H. pylori* infection and the incidence of GC. However, the gradual increase in antimicrobial resistance in *H. pylori* has encouraged researchers to not only identify new eradication strategies but also to improve the assessment of *H. pylori* susceptibility to the antimicrobials commonly used in treatment therapies. Constant surveillance of antibiotic resistance around the world is needed to improve *H. pylori* treatment. Recently, Li et. al. studied the antibiotic resistance pattern of *H. pylori* strains isolated from pediatric patients in Southwest China. The authors detected a very high resistance rate against commonly used drugs in therapy, in particular claritromycin (CLR) and metronidazole (AML) suggesting that the standard empiric CLR-based triple therapy should be avoided in the pediatric population in favor of bismuth quadruple therapy. In addition, most of the studies associated with *H. pylori* eradication in adult populations indicates that the standard empiric CLR-based triple therapy has poor efficacy (Macías-García et al., 2019). Therefore, gastroenterologists and physicians around the world must replace standard empiric therapy with a culture-based approach of isolate and assess for antibiotic susceptibility as a way to improve the efficacy of eradication (Li et al.).

*Helicobacter pylori* as many other bacteria adopt many self-defense mechanisms to evade the antimicrobial drugs and the host immune response. *H. pylori* has the ability to develop biofilm, to convert into a viable but non-culturable (VBNC) form, and to produce outer membrane vesicles (OMVs), spherical lipidic structures as a strategy to support its survival in the gastric mucosa (Cellini et al., 2005; Grande et al., 2015). Therefore, the identification of new strategies for the eradication of this microorganism represents an important goal. New synthesized Silver Ultra-Nanoclusters (SUNCs) characterized by an average size inferior to 5 nm and low toxicity on human cells, have been tested alone and in combination with metronidazole and clarithromycin, which have shown significant antimicrobial activity against *H. pylori*. In addition, SUNCs also displayed anti-biofilm activity which represents a potentially innovative and versatile approach for biofilm prevention (Grande et al.). Another novel successful eradication strategy is the identification of molecules that possess selective toxicity between pathogens and some colonizers of the human microbiota. *H. pylori* carbonic anhydrases (CAs) are involved in several physiological processes, like those related to the transport and supply of CO_2_ or ${\text{HCO}}_{3}^{-}$, pH homeostasis, or secretion of electrolytes. *H. pylori* CAs promotes the growth and host adaptation and are involved in acid tolerance in the gastric environment and modulation of toxin production (Angeli et al., 2018). Therefore, CAs represent suitable targets for microorganism eradication. In particular, the possibility of using CAIs (CA inhibitors) in association with probiotic bacterial strains could represent a novel anti-*H. pylori* therapeutic regiment that may contribute to limiting the antibiotic-resistance phenomenon (Campestre et al.).

In conclusion, the publications included in this Research Topic significantly advance our knowledge of the role that gastric microbiota might play in the development and progression of severe gastric diseases and can lead to major improvements in the prevention, diagnosis, and treatment of *Helicobacter*-associated conditions. They will help to better understand (i) the role of *H. pylori* in gastric inflammation and development of severe gastric diseases, particularly cancer; (ii) the virulence and pathogenesis expressed by the microorganism through the production of virulence factors, biofilm formation, the VBNC state, and release of OMVs; (iii) the impact of the microbiota on the development of gastric diseases as well as the effect of *H. pylori* infection on gastric microbiota composition and finally; and (iv) to identify innovative and efficient strategies for the eradication of *H. pylori* infection. We believe that this novel information will help to move forward knowledge of *H. pylori* infection.

## Author Contributions

Both authors listed have made a substantial, direct and intellectual contribution to the work, and approved it for publication.

## Conflict of Interest

The authors declare that the research was conducted in the absence of any commercial or financial relationships that could be construed as a potential conflict of interest.
